# Investigating 3D-printed disk compressing against skin for pain relief in intradermal infiltration anesthesia: a randomized controlled trial

**DOI:** 10.1186/s12871-023-02088-y

**Published:** 2023-04-28

**Authors:** Jiong Yu, Wenxuan Chen, Qianyuan Liu, Jingyi Mi

**Affiliations:** 1 Department of Sports Medicine, Wuxi 9Th People’s Hospital Affiliated to Soochow University, Wuxi, Jiangsu China; 2 Medical College, Soochow University, Suzhou, Jiangsu China

**Keywords:** 3D printing, Shotblocker, Needle pain, Local anesthesia, Pain relief

## Abstract

**Background:**

Pain intensity may be varied during the needle advancing through different skin layers, injection into the intradermal layer may exclude mixed pain from deeper planes. This study aimed to investigate whether compressing a three-dimensional (3D)-printed disk against the skin may relieve pain associated with intradermal injection of local anesthetic which mimics the skin test procedure.

**Methods:**

After institutional review board approval, 3D-printed disks with projections were designed for this study. Enrolled patients were randomized to receive either a disk compressing against the axillary skin during the intradermal injection of local anesthesia (compressing disk group) or an intradermal injection of local anesthesia without any compression (no compressing disk group). The primary outcomes were pain intensity (100-mm visual analog scale) and satisfaction (5-point Likert scale) as assessed by patients.

**Results:**

Ninety patients with American Society of Anesthesiologists I–II physical status receiving intradermal local anesthesia prior to an ultrasound-guided axillary approach were included. Eighty-seven patients completed the study, with 44 and 43 patients in disk and no disk groups, respectively. Pain scores were significantly different (*P* < 0.001) in compressing disk (median, 10; IQR, 5–20) and no compressing disk (median, 30; IQR, 20–40) groups. The median satisfaction score was 5 in both groups. No complications occurred during follow-up.

**Conclusion:**

Compressing a 3D-printed disk against the skin may reduce intradermal needle pain and offers an effective alternative for nerve block induction.

**Supplementary Information:**

The online version contains supplementary material available at 10.1186/s12871-023-02088-y.

## Introduction

In the setting of brachial plexus block for elective surgery, anesthetizing the local tissues is common for reducing this kind of pain intensity during the needle insertion process [1]. Paradoxically, the initial local injection still requires needle puncture, which incites acute needle pain. ShotBlocker® has been used to minimize the pain felt during intramuscular (IM) injection. In this application, the IM injection site is compressed using a blunt-tipped plastic disk with many projections. Although this method may relieve injection pain intensity [2–5], several studies have contradicting conclusions. Most randomized controlled trials have focused on its application in deep site injection (i.e., IM, spinal, and joint anesthesia). Moreover, pain intensity may be varied in different skin layers [6–8]. Under this concept, pain sensation may be mixed or overlapped during needle insertion and advancement, which may contribute to varied pain intensities that may influence the conclusions of these ShotBlocker®-related studies.

To the best of our knowledge, there are no published studies on the use of ShotBlocker® for intradermal needle insertion and injection pain relief. This study aimed to clarify whether a compressing device with projections may reduce the pain intensity associated with intradermal insertion and injection without advancing into deeper layers. However, this common device is still not available in China; therefore, we designed a three-dimensional (3D)-printed U-shaped ultraviolet (UV)-curable resin disk to confirm this hypothesis.

## Methods

### Study design and patients

This prospective randomized controlled study was approved by the ethics committee of our hospital on February 28, 2022 (approval number: LW2022004) and was registered in the Chinese Clinical Trial Registry (No. ChiCTR2200057324, date of first registration: 08/03/ 2022) before the first patient enrollment. All patients were informed of the purpose of the study and consented to participate. A flowchart was used for patient enrollment and allocation under the Consolidated Standards of Reporting Trials (CONSORT) guidelines.

### Inclusion and exclusion criteria

The patient inclusion criteria were 1) age ≥ 16 years Asian, 2) undergoing orthopedic surgery distal to the elbow, 3) American Society of Anesthesiologists (ASA) I–II physical status, 4) hospitalized patients who receiving intradermal injection of local anesthesia (intradermal lidocaine injection) prior to the ultrasound-guided axillary approach to brachial plexus block. The exclusion criteria were 1) emergency surgery, 2) analgesic medication within 6 h prior to injection, 3) anxiolytics or antidepressants medication status, impaired or unstable coagulation situation, 4) infection, 5) pre-existing skin pain at the site of needle insertion or of the involved limb, 6) allergic to local anesthesia, and 7) poor cooperation or verbal communication. Before the injection procedures, all patients were instructed to rate needle injection anxiety scores using a 5-point Likert scale [9], ranging from 0 (no anxiety) to 4 (strong anxiety). Those with Likert scores ≥ 3 points and with 20% higher than pre-injection (baseline) pulse rate modified from the literature [10] were also excluded. At last, a total of 106 hospitalized patients were assessed for study eligibility between March and June 2022, of which 90 met the inclusion criteria and were enrolled (CONSORT flow diagram). Eighty-seven patients completed the study analysis, and three individuals confused with the VAS score questions were finally excluded.

### Randomization and 3D-printed disk design

Using a Research Randomizer (www.randomizer.org) to generate two sets of 45 numbers with values of 1 or 2, each of two independent observers (WXC and QYL) received a certain set according to coin flip and sequentially enrolled patients according to the respective random number order. Number 1 represented the *compressing disk group*, *n* = 45, in which the 3D-printed disk with projections was compressed against the axillary skin during local anesthetic induction; number 2 represented the control (*no compressing disk group*), *n* = 45, which received regular intradermal injection of local anesthetic without any compressing devices.

**Fig. 1:**
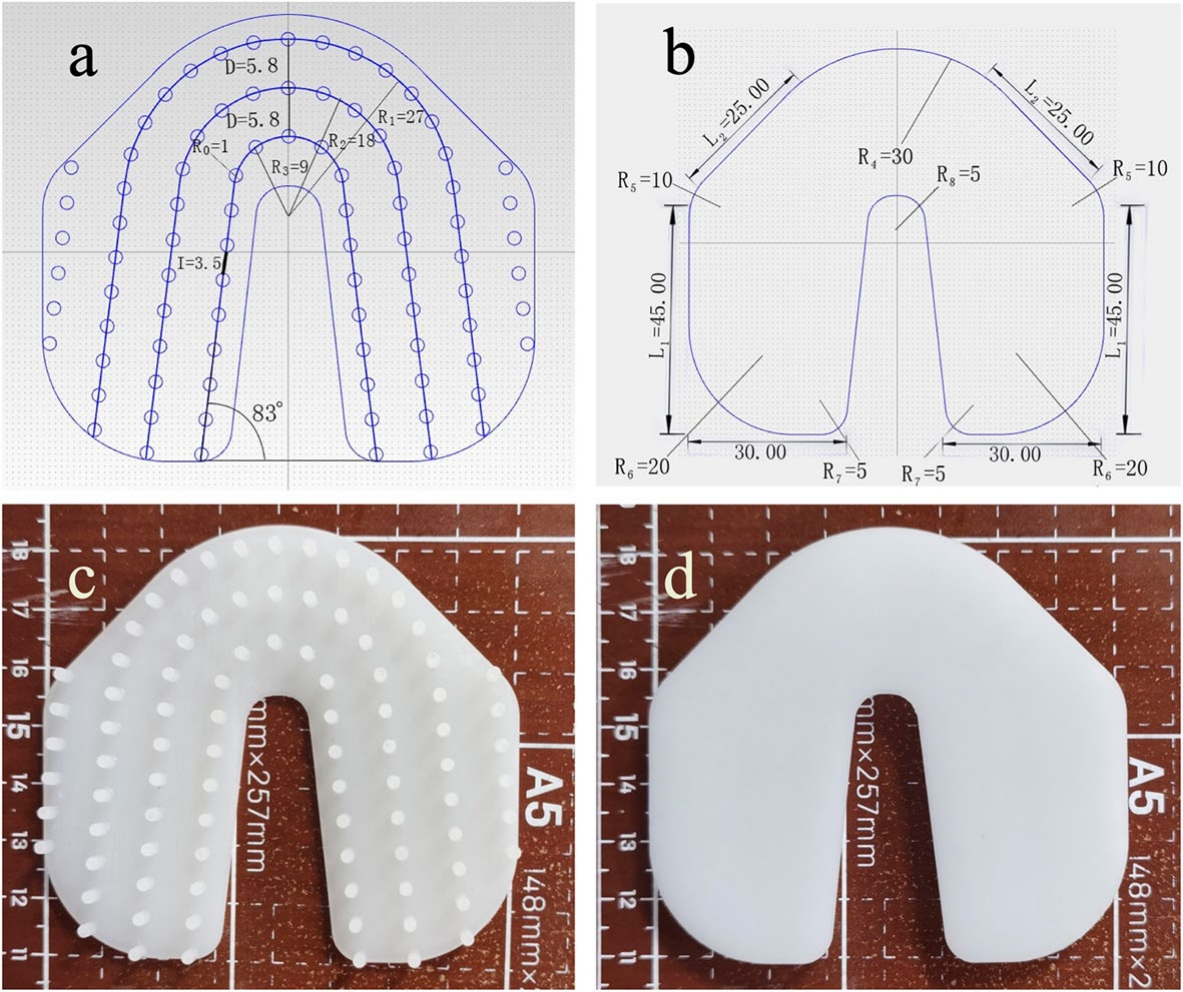
Three-dimensional (3D)-printed disk parameters and appearance. **a** curve projections with parameters. **b** shape data. **c** and **d** 3D-printed disk appearance with smooth and projection sides. All parameters are measured in mm

We used 3-matic (Materialize; Leuven, Belgium) to compress the disks according to the design (including shape, projection arrangement) of ShotBlocker®. The disks were printed using UV-curable resins into a smooth U shape with projections on one side. The detailed design parameters were as follows: radius for each projection cylinder (R_0_), 1 mm; height, 5 mm; and disk thickness, 1.5 mm. The projection curve data were as follows: outer projection curve radius (R_1_), 27 mm; middle projection curve radius (R_2_), 18 mm; and inner projection curve radius (R_3_), 9 mm. The interval between the two projections in the same curve, distance between arcs, side arm length, and lateral wing length were 3.5 mm, 5.8 mm, 45 mm, and 25 mm, respectively. The curve angle at the bottom was 83°. Regarding shape data, eight arcs were designed on the disk at the outer corners and fenestration bottom with radii of 30 mm (R_4_), 10 mm (R_5_), 20 mm (R_6_), 5 mm (R_7_), and 5 mm (R_8_). Their appearances are shown in Fig. 1. Each disk was recorded with its printed date, and the longevity was defined as 21 days to maintain the superior durable and flexible character of the UV-curable resins.

### Anesthesia

After confirming the inclusion and exclusion criteria in the operating room, all enrolled patients underwent pulse oximetry monitoring (fingertip pulse oximeter YX303, Jiangsu Yuyue Medical Equipment & Supply Co., Ltd., Jiangsu, China), without any analgesic or sedative medications. Sterile preparation was applied to the anesthesia site of the axilla, followed by a systematic ultrasound scan (SONIMAGE® MX1 Portable Ultrasound System with Linear Probe L11-3, Konica Minolta Healthcare Americas, Inc., Wayne, NJ, USA) to determine the optimal location for nerve block placement, and a point was marked for needle insertion. Each patient was instructed to keep the eyes shut and turn the head to the contralateral side during intradermal injection of local anesthesia prior to the nerve block. All anesthetic procedures were trained and conducted by three attending anesthetists. For the intervention group, the compressing disk was applied with projections against the axillary skin centered at the marked point (supplementary file “injection”). Subsequently, the skin wheal was raised by an intradermal injection of 3 mL lidocaine using a 22G block needle. The control group underwent the same procedure without the disk. An axillary brachial plexus block was performed using an ultrasound guide [11]. Anesthetic injection of 30 mL ropivacaine was observed with ultrasound surveillance.

### Outcome measurement

All patients consented to the study and were instructed in completing a 100-mm horizontal line visual analog scale (VAS) [9, 12] and a 5-point Likert scale [9] for needle-associated pain score and patient satisfaction (1, very dissatisfied; 2, dissatisfied; 3, neutral; 4, satisfied; 5, very satisfied). They were instructed to place a mark of “Ο” on the VAS scale line indicating their degree of intradermal injection pain and 5-point Likert scale sheet immediately after completion of the intradermal lidocaine injection. The VAS scale was scored by the observers measuring the distance from the low end of the scale to the mark by the respondent on the line. The pulse rate immediately before and after the intradermal lidocaine injection was documented. Anesthetists who administered the intradermal injection were instructed to assess the degree of usability of the disk using a 4-point scale: easy, moderate, difficult, or impossible [13]. To determine whether pain intensity may vary among individuals with different anxiety scores, three subgroups (0, strongly disagree; 1, disagree; 2, undecided) of anxiety intensity were formed. Side effects (allergic reactions) and unexpected events were investigated and described if they occurred. All these described data were documented according to the patient record paper sheets by the 3 anesthesiologists and then deliberately converted into anonymized electronical files by data collection group (WXC and QYL).

### Sampling and statistical analyses

The sample size was calculated using PASS (version 21.0.3; NCSS, Kaysville, UT, USA). With a significance level (alpha) of 0.05, using a one-sided two-sample unequal-variance t-test, group sample sizes of 36 and 36 achieved 99% power to reject the null hypothesis when the means of the compressing and no compressing disk groups were 27.91 and 18.85, respectively, with standard deviations of 10.54 and 8.45, respectively*.* Considering a 20% dropout rate, we finally allocated 90 patients, with 45 patients in each group. The Statistical Package for the Social Sciences (SPSS) version 26 (SPSS Inc., Chicago, IL, USA) was used for statistical analyses. Results are described as mean (standard deviation), median value (IQR), or proportion, as specified. The median (interquartile range [IQR]) pain scores and number (proportion) of patients with pain scores > 30 mm as moderate pain were calculated [14]. Parametric data (age, body mass index [BMI], proportion of patients with VAS > 30 mm) between the groups were compared using the independent Student’s t-test, and non-parametric data (VAS scores, Likert scores) were compared using the Mann–Whitney U-test. Categorical data (ASA status, sex) were compared using the χ^2^ test. Statistical significance was set at *P* < 0.05. To compare the distributions and density curves between the two groups, boxplot and violin plots were generated using http://www.bioinformatics.com.cn, a free online platform for data analysis and visualization.

## Results

**Fig. 2:**
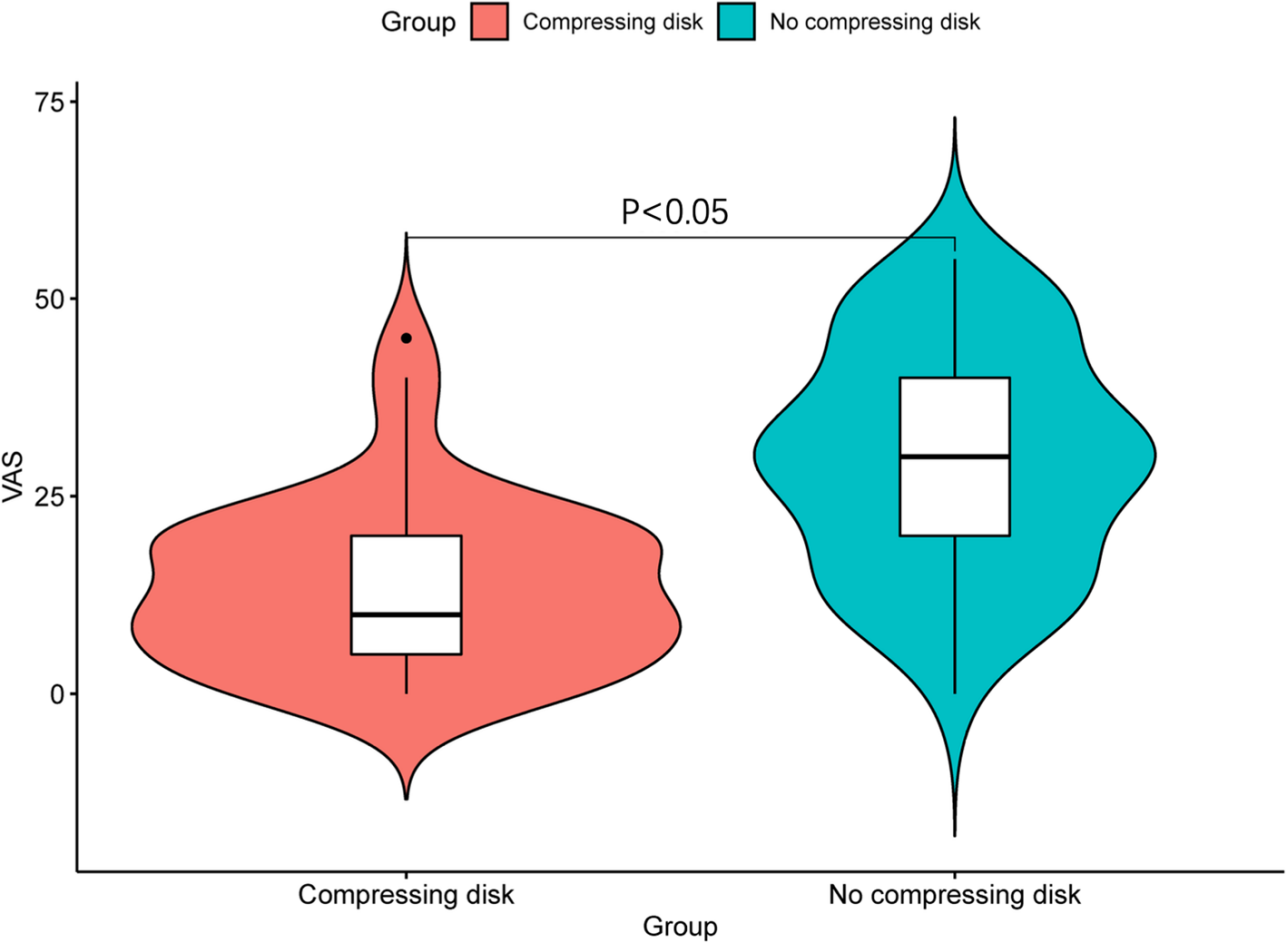
Violin and box/dot plot showing pain scores between the two groups which indicated that VAS pain score by using compressing disk is significantly lower than no compressing disk group. Lines in the boxes represent the median value, and boundaries of the boxes represent interquartile range. The black dot represents outlier. *P* value is shown in the lines above the plot

There were 44 patients randomized to the compressing disk group, 43 in no compressing disk group with mean age of 44 ± 13 and 46 ± 13 years old respectively. Sixty men and twenty-seven women were included in this study. BMI and ASA classification distributions were comparable between the two groups with no data significant (Table 1). Despite the differences in pain intensity between the two groups, patient satisfaction in both groups was similar, with a median value of 5 points (IQR, 4–5). Pulse rates before and after intradermal lidocaine injection were not significantly different between the two groups (*P* > 0.05). The median pain severity in the intervention group (red violin plot) was 10 mm (IQR, 5–20), which was significantly lower than that in the control group (green violin plot), whose median pain score was 30 (IQR, 20–40), as shown in Fig. 2 and Table 1 (*P* < 0.001). The VAS scores > 30 mm were 0.068 (3/44) and 0.605 (26/43) in the compressing and no compressing disk groups (*P* < 0.001), respectively, a statistically significant difference. Observed, from the boxplot and violin plot, VAS pain scores are distributed more concentrated near the median value in the compressing disk group than no compressing group. The pain scores between the two groups were significantly different in both anxiety subgroups (*P* < 0.001). The median usability of the compressing disk was 1 point, indicating easy use in the procedure. All patients were observed and followed up at 8 h without unexpected events or any axillary site complications.

**Table 1:** Comparison between patients with and without compressing disk intervention during the intradermal lidocaine injection. Values are expressed as mean (standard deviation), median (interquartile range), or proportion. VAS scores were significantly higher in no compressing disk compared to the intervention group, anxiety Likert scores, pulse rate and patient satisfactory Likert scores were no significantly different

	Compressing disk group	No compressing disk group	*P* value^*,†^
Sample size, n	44	43	
Sociodemographic characteristics			
Mean age (SD) in years	44 (13)	46 (13)	0.291
Sex, n (%)			0.529
Male	30 (68.28)	30 (69.77)	
Female	14 (31.82)	13 (30.23)	
Mean BMI (SD) in (kg/m^2^)	24.16 (3.32)	23.83 (2.48)	0.298
Local anesthesia characteristics			
ASA classification, n (%)			0.404
I	36 (81.8)	37	
II	8 (18.2)	6	
Anxiety Likert scale			
0	7	16	
1	19	19	
2	18	8	
Mean pulse rate prior to the injection (SD)	78 (13)	76 (13)	0.209
Mean pulse rate after the injection (SD)	79 (15)	75 (13)	0.094
Median VAS (interquartile range) in mm	10 (5–20)	30 (20–40)	< 0.001
VAS score over and below 30 mm			< 0.001
VAS score ≥ 30 mm, n (%)	3 (6.82)	26 (60.47)	
VAS score < 30 mm, n (%)	41 (93.18)	17 (39.53)	
Patient satisfactory Likert scale (interquartile range)	5 (4–5)	5 (4–5)	0.231
Disk usability Likert scale (interquartile range)	1 (1–2)	NA	NA

*Abbreviations*: *SD* Standard deviation, *BMI* Body mass index, *ASA* American Society of Anesthesiologists, *VAS* Visual analog scale, *NA* not applicable

^*^P value comparing the compressing and no compressing disk groups

^†^The t-test was used to compare means, Mann–Whitney U-test was used to compare medians, and chi-squared test was used to compare proportions

## Discussion

The findings of this study clearly indicate that the 3D-printed compressing disk may reduce the pain intensity associated with axillary intradermal needle insertion and injection. Using physical methods to relieve pain may improve patients’ quality of life by minimizing painful sensation and reducing the use of analgesics [15]. To specify the moderate needle pain intensity as a watershed, we referred the classic literature which concluded VAS = 30 mm should be the moderate pain [14], and the study showed pain-relief effect of the 3D printed disk with lesser moderate and severe pain. Several studies have reported that manual pressure during IM injection may reduce pain [16–18]. Current physical products for pain relief, such as ShotBlocker®, based on the gate control theory described by Melzack and Wall [19], have blunt projections to stimulate larger afferent nerve fibers when compressing around the injection site, thus inhibiting pain perception by exerting an inhibitory effect on afferent sensory fibers by activating cells within the substantia gelatinosa in the dorsal horn of the spinal cord. Applying ShotBlocker® for local anesthesia in general places has proven effective for pain relief [4, 5]. A recent meta-analysis concluded that the application of ShotBlocker® can reduce IM injection pain in adults [20], although two additional randomized controlled trials in IM injection and spinal anesthesia reported contradicting results [13, 21] These different study results may be affected by heterogeneity of the injection site, volume, and depth [22]. Furthermore, pain intensity could vary with the depth of the injection site [6–8, 23] When inserting the needle to the muscular level, it penetrates the intradermal and subdermal layers in route, and pain sensation may be mixed or overlapped. Meanwhile, patients with extreme injection anxiety, who may have higher VAS scores than their counterparts, should be excluded [20]. The results showed that patient anxiety might not be related to needle pain intensity, especially in individuals with Likert scores of 0–2.

To minimize these heterogeneities, we designed the current study focusing on the intradermal level of the axilla as the first layer of the injection site (to exclude site and depth variables, in contrast to previous related studies), a uniform 3-mL lidocaine volume, and unregulated injection speed (because it has no effect on pain scores according to the study) [22]. The irregular plane of the axilla may be considered unfeasible for placing a flat disk; however, all intervention cases demonstrated easy manipulation for placing the flexible compressing device, even in patients with a lower BMI.

Considering the heterogeneity of pain sensation, especially injection anxiety, which may affect pain scores and satisfaction [24, 25], it is necessary to exclude extremely anxious patients. Although the Injection Anxiety Scale–Anxiety and State-Trait Anxiety Inventory Form can be a promising measurement tool, items 8 and 20 of the scale may be time-consuming due to its lengthy administration time in the setting of anesthesia procedures [26]. A 5-point scale for measuring anxiety has been used for cannulation [9], and we used it during trial enrollment as a swift way to exclude patients with injection anxiety that may influence the pain score. Interestingly, though pain intensity is different among two groups, satisfaction scale is not statistically significant.

It was demonstrated that stressor (e.g. needle pain) can induce difference in vital signs by stimulating the sympathetic nervous system [27]. In addition, anxiety may physiologically affect pulse rate and pain threshold [28–30], for these reasons, 20% increased pulse rate combined with anxiety Likert scores ≥ 3 points in this study was applied as an supplementary indicator to exclude those potential biased VAS scores; at the same time, we want to study if pulse rate may be affected by the 3D printed disk compressing in Asian population. The results show there was no significant differences before and after the injection, which is consist with the literatures [31, 32].

The skin wheal for local anesthesia in spinal block has recently been considered a controversial topic [33]; however, this procedure is still common, especially in ultrasound-guided regional anesthesia [1, 34–37]. In this study, we preferred to raise the skin wheal as a landmark for anatomical discrimination. We did not apply a compressing disk for nerve block under the ultrasound-guided procedure because of the obstacle effect of the disk, especially when it was performed by only one person.

### Strengths and limitations of this study

As strengths, the present study used randomization and uniform application sites, insertion layer, needle gauge, and patient conditions to investigate whether a compression disk with projections may reduce pain intensity during intradermal infiltration anesthesia. Our results supported compression disk use.

This study has certain limitations. The study was conducted at a single center with a relatively small patient sample. Furthermore, a placebo group was not included because former related studies had designated the flat side of the disk as a placebo intervention [4, 13]; however, the pain intensity may still have been reduced by manual pressure [16]. Future studies should investigate whether placing the flat side of the disk and compressing it against the skin leads to similar outcomes as using the projection side of the disk. Those with higher anxiety scores (> 3) and ASA ≥ III were excluded, and the corresponding pain intensity could not be assessed due to selection bias; thus, the relationship between pain and anxiety was not explored. Directing the patients’ heads toward the opposite side during intradermal lidocaine injection had a minor blinding effect because patients were able to feel the device compressing their skin, and it was almost impossible to blind the patient and anesthetist to the procedure.

In conclusion, this prospective randomized study showed that a 3D-printed UV-curable resin-compressing disk may relieve needle insertion and injection pain for axillary intradermal needle puncture during intradermal lidocaine injection. This is a safe, convenient, and effective physical analgesic method prior to nerve block anesthesia.

## Supplementary Information

**Additional file 1.**

**Additional file 2.**

**Additional file 3.**

## Data Availability

All data generated or analysed during this study are included in this published article and its supplementary information files.
