# Supplementary figures and images for: Investigating 3D-printed disk compressing against skin for pain relief in intradermal infiltration anesthesia: a randomized controlled trial
Source: BMC Anesthesiol. 2023 Apr 28;23:144. doi: 10.1186/s12871-023-02088-y (PMC10148480; doi:10.1186/s12871-023-02088-y)

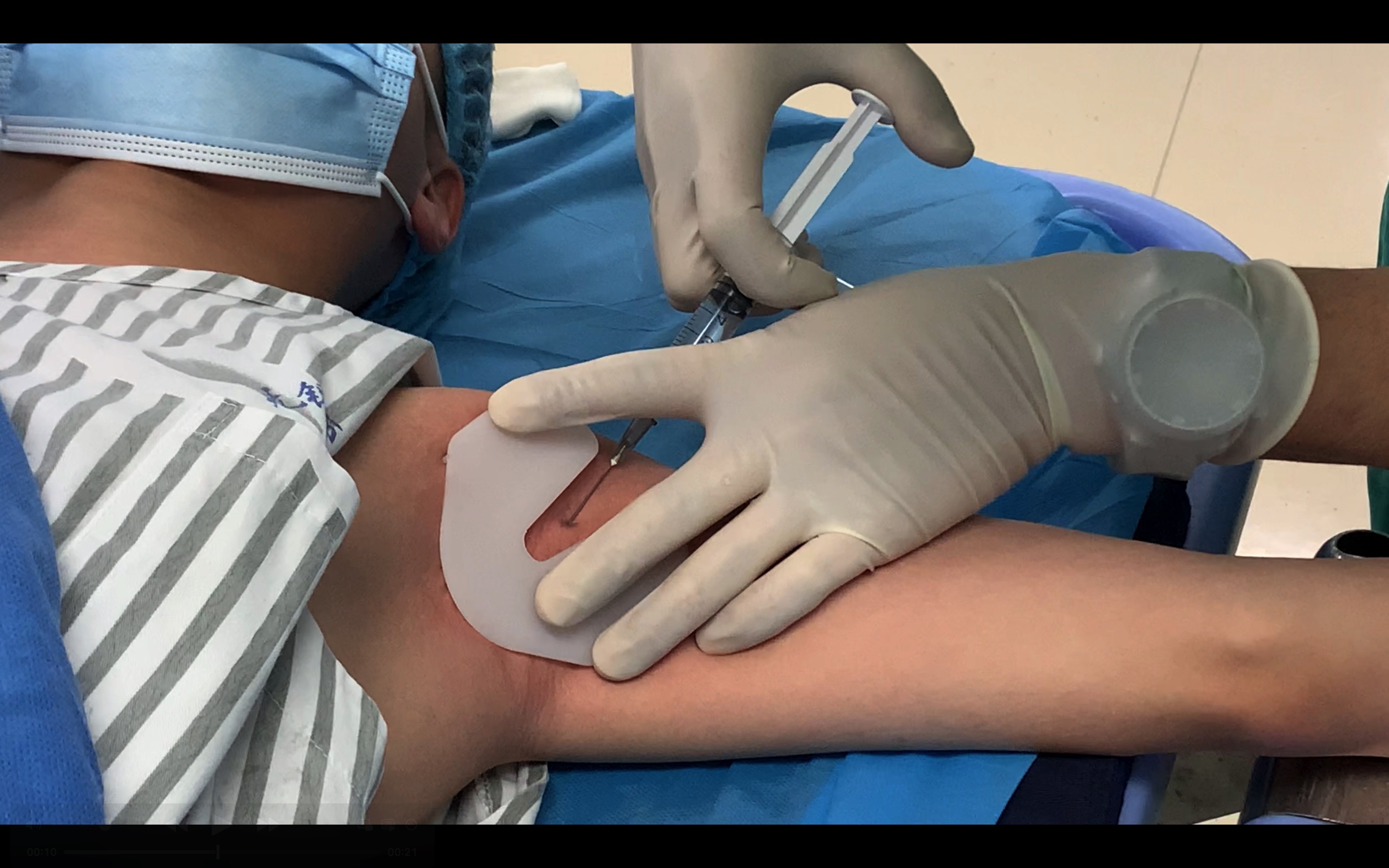

Supplement: Supplementary file 1 — Additional file 1. [file 12871_2023_2088_MOESM1_ESM.jpg]
