# Supplementary material for: Investigating 3D-printed disk compressing against skin for pain relief in intradermal infiltration anesthesia: a randomized controlled trial
Source: BMC Anesthesiol. 2023 Apr 28;23:144. doi: 10.1186/s12871-023-02088-y (PMC10148480; doi:10.1186/s12871-023-02088-y)
Supplement: Supplementary file 2 — Additional file 2. [file 12871_2023_2088_MOESM2_ESM.zip › attached files/electronical version/LQY.pdf]

ID	Group	Gender	Age	BMI	ASA	Pre. PR	Post. PR	4-point Likert Anxiety	VAS	5-point Likert satisfaction	feasibility
202206023	1	m	20	16.53	I	70	78	2	0	5	2
202206013	2	m	25	21.26	I	80	75	1	40	5	NA
202206275	1	m	33	29.39	I	77	85	2	10	4	2
202206296	1	m	38	28.07	I	74	77	1	10	5	1
202206254	2	m	41	22.86	I	66	65	2	10	5	NA
202206255	1	f	34	19.43	I	112	124	2	40	4	1
202206316	1	m	73	20.76	I	63	61	1	5	5	1
202206058	2	m	33	21.8	I	63	67	1	30	4	NA
202206444	1	m	54	24.22	I	86	86	1	20	5	2
202206502	2	m	49	24.22	I	69	72	1	45	5	NA

202206285	2	m	53	22.31	I	65	60	2	15	5	NA
202206503	1	m	47	22.77	I	85	80	0	0	5	1
202206594	1	m	46	26.2	I	84	69	1	0	5	1
202206511	2	m	58	22.72	I	68	69	1	30	5	NA
202206497	1	f	29	16.73	I	86	84	2	25	5	2
202206539	2	f	49	26.04	I	79	72	0	35	5	NA
202206547	2	m	23	26.32	I	68	67	1	35	5	NA
202206542	1	m	53	25.54	I	60	58	0	5	5	1
202206435	1	m	43	26.23	II	77	78	2	2	4	1
202206512	1	m	32	31.67	I	88	77	1	2	5	1
202206504	1	f	44	22.77	I	93	85	2	15	5	2
202206717	1	m	50	25.71	II	76	77	0	3	5	1
202206614	1	m	29	18.13	I	65	66	1	10	5	1

202205317	1	m	38	22.86	I	97	99	1	20	5	1
202206739	1	f	49	26.67	II	72	75	0	10	5	2
202206670	2	m	57	21.48	I	72	73	0	20	5	NA
202206470	2	m	66	21.77	I	76	78	0	30	5	NA
202206750	1	m	41	27.18	I	87	90	0	10	5	1
202206723	2	m	33	24.07	I	94	97	2	15	4	NA
202206716	2	f	32	21.3	I	76	71	1	10	4	NA
202206384	2	f	56	22.04	II	95	100	1	50	5	NA
202206700	2	m	33	23.94	I	60	63	1	10	5	NA
202205504	1	f	48	23.87	I	63	71	1	20	4	1
202206795	1	m	54	22.85	I	78	72	1	15	5	2
202206180	1	m	39	26.57	I	74	72	1	20	4	1
202206783	2	m	16	29.39	I	69	63	0	30	5	NA

202206775	2	f	41	22.86	I	68	72	1	20	5	NA
202206805	2	m	67	23.67	I	80	87	0	20	4	NA
202206347	1	m	32	24.22	I	93	88	1	10	5	1
202206765	1	f	54	26.71	I	67	71	1	10	5	1
202206798	1	m	75	24.8	II	84	92	1	10	5	1
202206600	2	f	63	23.87	I	76	70	1	10	4	1
202206834	2	f	43	23.88	I	84	67	0	25	5	NA
202206801	2	m	24	22.86	I	66	72	0	30	5	NA
202206843	1	f	72	29.3	II	98	81	0	20	5	1
