# Supplementary material for: Investigating 3D-printed disk compressing against skin for pain relief in intradermal infiltration anesthesia: a randomized controlled trial
Source: BMC Anesthesiol. 2023 Apr 28;23:144. doi: 10.1186/s12871-023-02088-y (PMC10148480; doi:10.1186/s12871-023-02088-y)
Supplement: Supplementary file 2 — Additional file 2. [file 12871_2023_2088_MOESM2_ESM.zip › attached files/electronical version/CWX.pdf]

ID	Group	Gender	Age	BMI	ASA	Pre. PR	Post. PR	4-point Likert Anxiety	VAS	5-point Likert satisfaction	feasibility
202204777	1	m	67	25.39	II	67	65	1	25	5	2
202204688	2	m	57	21.51	II	108	106	0	50	3	NA
202204680	1	f	31	20.7	I	70	84	2	14	4	1
202205753	2	m	51	21.01	I	64	70	1	50	4	NA
202205722	1	m	34	24.22	I	72	80	2	35	4	1
202205741	2	f	63	20.81	II	78	72	2	44	3	NA
202204533	1	m	55	24.22	I	85	90	2	45	3	2
202205718	1	f	52	20.69	II	70	72	2	20	4	1
202205844	2	f	35	22.6	I	92	97	2	55	5	NA
202205751	2	m	33	25.95	I	98	68	1	30	5	NA

202205320	1	f	48	25.39	I	90	120	1	24	4	1
202206192	1	m	17	18.94	I	95	85	2	20	5	NA
202206153	1	m	45	25.5	I	67	62	2	21	4	1
202206188	2	m	55	20.76	I	67	72	1	20	4	NA
202206163	1	m	32	25.25	I	61	58	0	1	5	1
202205734	1	f	44	24.89	I	83	70	1	9	3	2
202206049	2	m	55	22.49	I	60	57	0	33	5	NA
202206199	2	m	34	28.37	I	86	64	0	10	5	NA
202206024	1	m	54	21.87	I	60	59	1	19	5	1
202206128	2	m	41	26.73	I	81	89	0	0	5	NA
202205205	2	f	35	22.04	I	60	57	2	40	5	NA
202206248	2	f	52	25.39	I	71	72	2	40	3	NA
202206399	2	m	59	25.7	I	73	79	1	50	2	NA

202205742	1	m	27	20.76	I	96	79	2	15	3	3
202206287	1	m	57	25.91	II	79	80	2	0	5	2
202206388	2	m	26	24.49	I	65	70	2	50	2	NA
202206266	1	f	60	27.04	I	63	62	2	10	5	1
202205183	2	m	56	22.31	I	74	72	1	30	4	NA
202206268	2	m	58	19.75	I	77	75	0	30	4	NA
202206366	2	f	27	23.44	I	85	67	1	30	3	NA
202206284	2	f	63	23.43	II	117	110	1	28	4	NA
202206247	2	m	54	25.95	II	66	66	1	30	4	NA
202205646	2	m	56	26.12	II	87	79	1	40	4	NA
202205757	1	f	61	33.87	II	54	41	0	15	5	2
202206086	1	f	52	23.73	I	70	72	2	20	4	1
202206333	2	m	50	31.25	I	56	82	0	10	5	NA

202206253	2	m	46	21.6	I	82	75	2	10	5	NA
202206029	2	m	52	25.47	I	80	72	0	15	5	NA
202206385	2	f	48	24.78	I	80	99	0	50	5	NA
202206420	2	m	44	23.88	I	75	71	0	10	5	NA
202206105	1	m	23	27.16	I	97	108	2	5	5	1
202206449	1	f	48	20.4	I	86	87	2	20	4	1
202206211	2	f	53	26.94	I	62	55	1	20	5	NA
202206401	1	m	52	25.25	I	80	93	1	5	5	1
202206206	1	m	26	26.73	I	52	50	1	5	5	2
